# Supplementary material for: Elevating fungal care: bridging Brazil’s healthcare practices to global standards
Source: Microbiol Spectr. 2025 Mar 10;13(4):e02112-24. doi: 10.1128/spectrum.02112-24 (PMC11960063; doi:10.1128/spectrum.02112-24)
Supplement: Table S1 — Access to treatment tools and therapeutic drug monitoring of participating institutions in Brazil and Europe. [file spectrum.02112-24-s0001.docx]

## Elevating Fungal Care: Bridging Brazil's Healthcare Practices to Global Standards

**Supplementary tables**

**Supplementary table 1.** Access to treatment tools and therapeutic drug monitoring of participating institutions in Brazil and Europe.

|  | **Brazil** | | **Europe** | | **p value** |
| --- | --- | --- | --- | --- | --- |
|  | ***n*** | ***%*** | ***n*** | ***%*** |  |
| **Available antifungals** |  |  |  |  |  |
| Amphotericin B, any | 69/96 | 71.9 | 337/388 | 86.9 | <0.001 |
| *Amphotericin B deoxycholate* | 47/96 | 49.0 | 159/388 | 41.0 | 0.157 |
| *Amphotericin B lipid complex* | 35/96 | 36.5 | 124/388 | 32.0 | 0.401 |
| *Amphotericin B liposomal* | 33/96 | 34.4 | 301/388 | 77.6 | <0.001 |
| Echinocandins | 53/96 | 55.2 | 346/388 | 89.2 | <0.001 |
| *Anidulafungin* | 26/96 | 27.1 | 251/388 | 64.7 | <0.001 |
| *Caspofungin* | 14/96 | 14.6 | 335/388 | 86.3 | <0.001 |
| *Micafungin* | 42/96 | 43.8 | 254/388 | 65.5 | <0.001 |
| Triazoles | 90/96 | 93.8 | 363/388 | 93.6 | 0.945 |
| *Fluconazole* | 89/96 | 92.7 | 362/388 | 93.3 | 0.837 |
| *Isavuconazole* | NR | NR | 235/388 | 60.6 | - |
| *Itraconazole* | 63/96 | 65.6 | 313/388 | 80.7 | 0.002 |
| *Posaconazole* | 13/96 | 13.5 | 300/388 | 77.3 | <0.001 |
| *Voriconazole* | 43/96 | 44.8 | 346/388 | 89.2 | <0.001 |
| Flucytosine | NR | NR | 193/388 | 49.7 | - |
| Terbinafine | NR | NR | 202/388 | 52.1 | - |
| **Therapeutic drug monitoring** | 9/96 | 9.4 | 250/388 | 64.4 | <0.001 |
| Flucytosine | NR | NR | 122/388 | 31.4 | - |
| Itraconazole | 2/96 | 2.1 | 172/388 | 44.3 | <0.001 |
| Posaconazole | 1/96 | 1.0 | 209/388 | 53.9 | <0.001 |
| Voriconazole | 7/96 | 7.3 | 250/388 | 64.4 | <0.001 |

**NR**, not reported

Data from Brazil were previously included in a collective publication alongside data from other Caribbean and Latin American countries. ^1^ The European data, in its present format, have been published separately elsewhere. ^2^

1. Falci DR, Pasqualotto AC. Clinical mycology in Latin America and the Caribbean: A snapshot of diagnostic and therapeutic capabilities. *Mycoses* 2019; **62**(4): 368-73.

2. Salmanton-Garcia J, Hoenigl M, Gangneux JP, et al. The current state of laboratory mycology and access to antifungal treatment in Europe: a European Confederation of Medical Mycology survey. *Lancet Microbe* 2023; **4**(1): e47-e56.
